# Extra-territorial movements differ between territory holders and subordinates in a large, monogamous rodent

**DOI:** 10.1038/s41598-017-15540-0

**Published:** 2017-11-10

**Authors:** Martin Mayer, Andreas Zedrosser, Frank Rosell

**Affiliations:** 1 Department of Natural Sciences and Environmental Health, University College of Southeast Norway, Bø i Telemark, Norway; 2 Department of Integrative Biology, Institute of Wildlife Biology and Game Management, University of Natural Resources and Life Sciences, Vienna, Austria

## Abstract

Territorial animals carry out extra-territorial movements (forays) to obtain pre-dispersal information or to increase reproductive success via extra-pair copulation. However, little is known about other purposes and spatial movement patterns of forays. In this study, we GPS-tagged 54 Eurasian beavers (*Castor fiber*), a year-round territorial, monogamous mammal, during the non-mating season. We investigated forays in territory-holding breeders (dominants) and non-breeding (subordinate) family members. Twenty of 46 dominant individuals (44%), and 6 of 10 subordinates (60%) conducted forays. Generally, beavers spent between 0 and 11% of their active time on forays, travelled faster and spend more time in water when on forays compared to intra-territorial movements, suggesting that forays are energetically costly. Further, beavers in smaller territories conducted more forays. Possibly, smaller territories might not have sufficient resources and thus dominant individuals might conduct forays to assess possibilities for territory expansion, and potentially for foraging. Generally, besides territory advertisement (e.g. via scent-marking), forays might serve as an additional mechanism for territory owners to assess neighbours. Subordinates spent more time on forays, moved greater distances and intruded into more territories than dominant individuals did, suggesting that they prospected to gain information on the population density and available mates before dispersal.

## Introduction

Animals are territorial when the benefits of holding a territory (defined as the exclusive access to limited resources, such a food, mating partners and shelter) exceed the total costs of territory defence^1^. Spatio-temporal movements outside of an animal’s or a breeding pair’s territory are defined as extra-territorial movements (hereafter forays)^2,3^. Forays are important for ecological processes such as gene flow^4,5^ and dispersal^6^, and are common in vertebrates including fish^2^, birds^7–9^, and mammals^10–12^. Generally, three types of forays are distinguished: (1) Adults seeking extra-pair copulations^12–14^. This type of foray relies on information about neighbours^15^ and habitat structure^8^ and is usually limited to the reproductive season^16^; (2) Forays by subordinate individuals (non-breeders) to gain experience and information about dispersal opportunities^16–19^; and (3) Forays to increase foraging success. For example, wolves (*Canis lupus*) conducted forays during periods of low food availability^19^ and feral cats (*Felis catus*) in Australia carried out forays into recently burned areas, probably providing them with foraging opportunities due to the availability of vulnerable prey^20^. However, apart from gaining foraging opportunities, little is known about the role of forays of year-round territory holders outside of the mating season, and especially about the movement patterns during forays in general^3^.

The frequency of forays can vary with demographic parameters. In birds, forays are often skewed towards males^8,21^. In song sparrows (*Spizella pusilla*) and reed buntings (*Emberiza schoeniclus*) older males conducted more forays than younger ones^22,23^. Because forays generally have other purposes than intra-territorial movements (movements inside the home territory), e.g. prospecting for extra-pair copulations, their movement patterns can also differ. For example, dispersing red foxes (*Vulpes vulpes*) moved faster and straighter when outside of their own territory and avoided contact with territorial adults, and adult territory owners often intruded into neighbouring core areas during forays^3^.

In this study we investigated forays outside the mating season in a large, monogamous, semi-aquatic rodent, the Eurasian beaver (*Castor fiber*, hereafter beaver). Beavers are nocturnal, live in family groups and ecologically are very similar to the North American beaver (*C. canadensis*)^24,25^. They are highly territorial and defend territories year-round against conspecifics^26^. Territory occupancy is advertised at the territory borders via scent-marking^26^. Beavers are central place foragers^27^ that feed on deciduous woody plants, herbs, and aquatic vegetation^28,29^. Mating in both species takes place in January and February^24^. Extra-pair copulations have been recorded in a North American beaver population^30^, suggesting that forays occur during the mating season, whereas there is little evidence for extra-pair copulations in Eurasian beavers^31,32^. Non-breeding family members ≥ 1 year old (hereafter subordinates) typically disperse at age 1.5^33^ to 3.5 years old^34^, but can delay dispersal up to age 7^34^. An increased age at dispersal has been related to high population densities^33,35^ and Mayer, *et al*.^34^ suggested that individuals can perceive changes in population density before initiating dispersal. Forays may be the mechanism to evaluate population density levels and there is evidence for pre-dispersal prospecting in beavers. For example, 3 of 8 subordinates made forays before dispersal in a North American beaver population^36^, and 3 of 9 subordinates carried out pre-dispersal forays ranging between 1.5 and 15 km in a Swedish beaver population^33^. However, to our knowledge there is no information about forays of territory-holding breeders (hereafter dominants) or movement patterns during forays in general.

**Figure 1:**
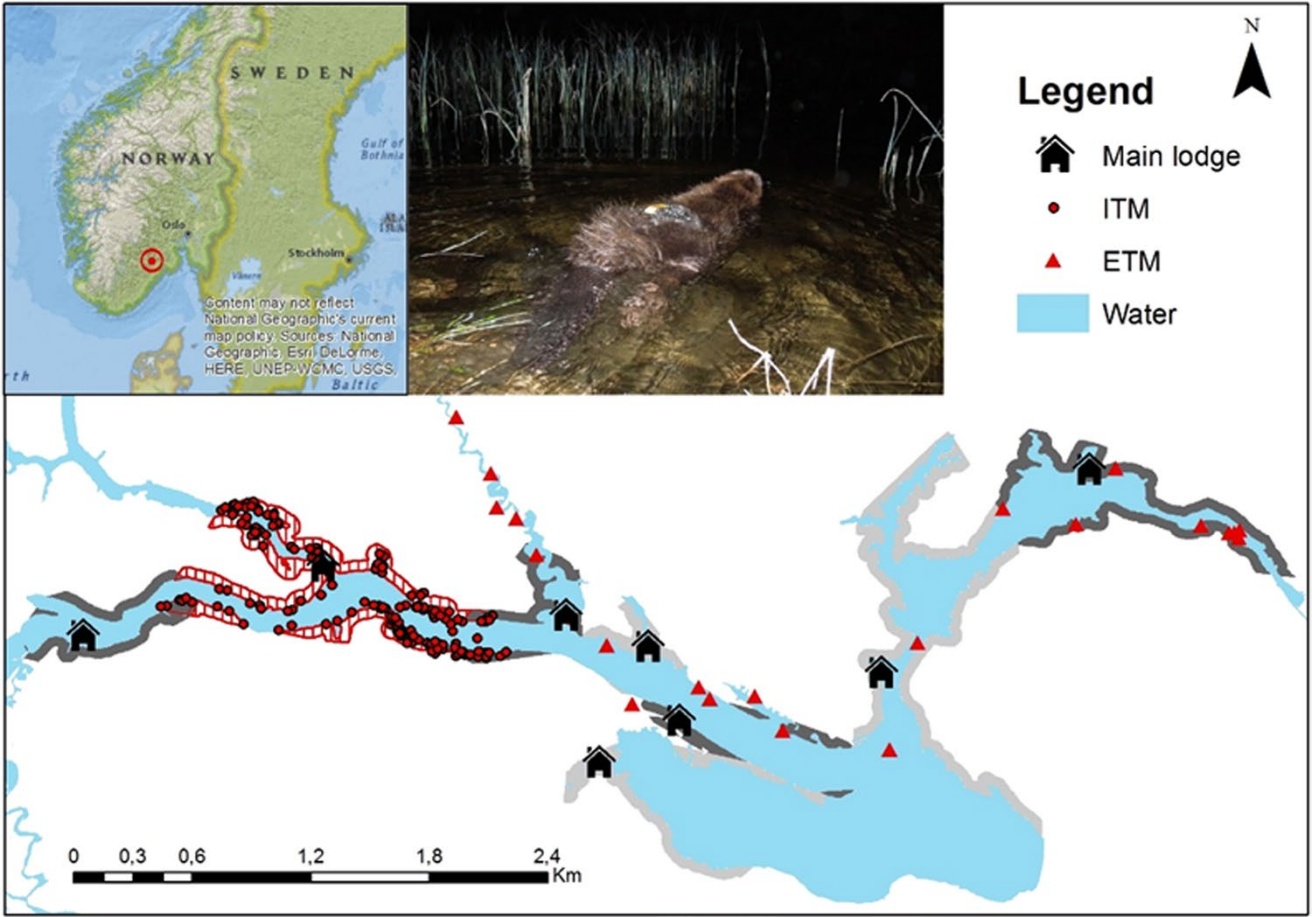
The location of our study area in southeast Norway (top right), and our study species, the Eurasian beaver, with a GPS on its back (top middle). The main map shows exemplary GPS data from a subordinate individual who conducted extra-territorial movements (ETM, red triangles) through five different territories (shown with grey shadings). The territory is shown in red hatching and intra-territorial GPS positions (ITM) are shown as red dots. The figure was created using ArcMap 10.1 (Esri, Redlands, CA, USA, http://www.esri.com/arcgis/about-arcgis). Source of small map (top right): National Geographic, Esrif DeLorme.

Here we investigated foray patterns by dominant and subordinate beavers during the non-mating season in a beaver population in southeast Norway (Fig. 1). We could not investigate forays during the mating season in winter due to difficulties in observing, capturing and attaching GPSs to often ice-bound beavers at this time of the year. We hypothesized that foray patterns are related to an individual’s social status. We predicted that dominant territory owners would conduct forays into adjacent neighbouring territories to assess the potential for territory expansion or to gain foraging opportunities, and that subordinates would conduct more and longer forays to gain pre-dispersal information. Further, we hypothesised that foray patterns would be related to territory size, individual age, and season. We predicted that beavers in smaller territories would conduct more forays because smaller territories potentially lack sufficient resources^37^, and that younger individuals would conduct more forays than older ones because older individuals (>8 years) have been shown to senesce^38,39^, which might inhibit them from conducting presumably costly forays. Additionally, seasonal differences might affect the resource availability, which could lead to altered foray frequencies between spring and fall. Finally, we hypothesized that movement patterns would differ between intra-territorial movements and forays, and predicted that individuals would travel at a greater speed and spend less time on land during forays compared to intra-territorial movements.

## Results

**Figure 2:**
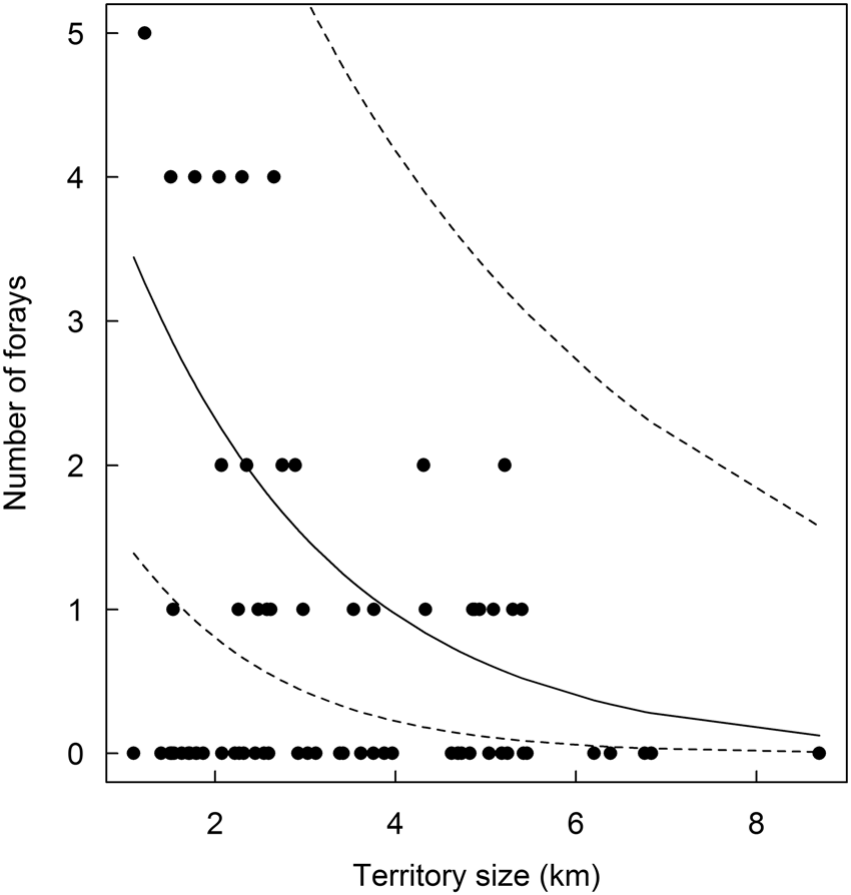
Predicted relationship (solid line) between the territory size and the number of forays of 54 GPS-tagged Eurasian beavers in southeast Norway (2009–2016). Dashed lines represent upper and lower confidence intervals.

We GPS tagged a total of 54 individuals: 46 dominant individuals consisting of 23 females (7 were tagged multiple times) and 24 males (6 were tagged multiple times), and 10 subordinate (4 females and 6 males) beavers. Two individuals were first trapped as subordinate and then as a dominant individual. Of the 54 GPS tagged individuals, 25 (46.3%) carried out forays, while 20 of 46 (43.5%) dominant beavers and 6 of 10 (60%) subordinates conducted forays. Beavers spent between 0.00 and 10.63% of their active time on forays (mean ± SD: 1.47 ± 2.76%, median: 0%). They conducted between zero and five forays during the GPS sampling period (mean ± SD: 0.71 ± 1.22, median: 0); on average 0.59 ± 0.97 (median: 0, range: 0–4.4) per week. The number of forays was best explained by the territory size (Tables 1 and S1) with individuals in smaller territories conducting more forays compared to larger ones (Fig. 2).

**Table 1:** Effect size (β), standard error (SE), and lower (LCI) and upper (UCI) 95% confidence intervals of explanatory variables for the number of extra-territorial movements of 54 GPS-tagged Eurasian beavers in southeast Norway (2009–2016).

Variable	β	SE	LCI	UCI
**Territory size (km)**	**−0.425**	**0.129**	**−0.682**	−**0.167**
Age	−0.098	0.086	−0.270	0.074
Season (spring)	−0.371	0.325	−1.018	0.277
GPS positions	0.000	0.001	−0.001	0.002
Status (subordinate)	−0.058	0.460	−0.975	0.860

We performed model averaging of best models (∆AIC_c_ < 4) to estimate the effect size of each variable. Informative parameters are presented in bold.

### Differences between forays and intra-territorial movements

**Figure 3:**
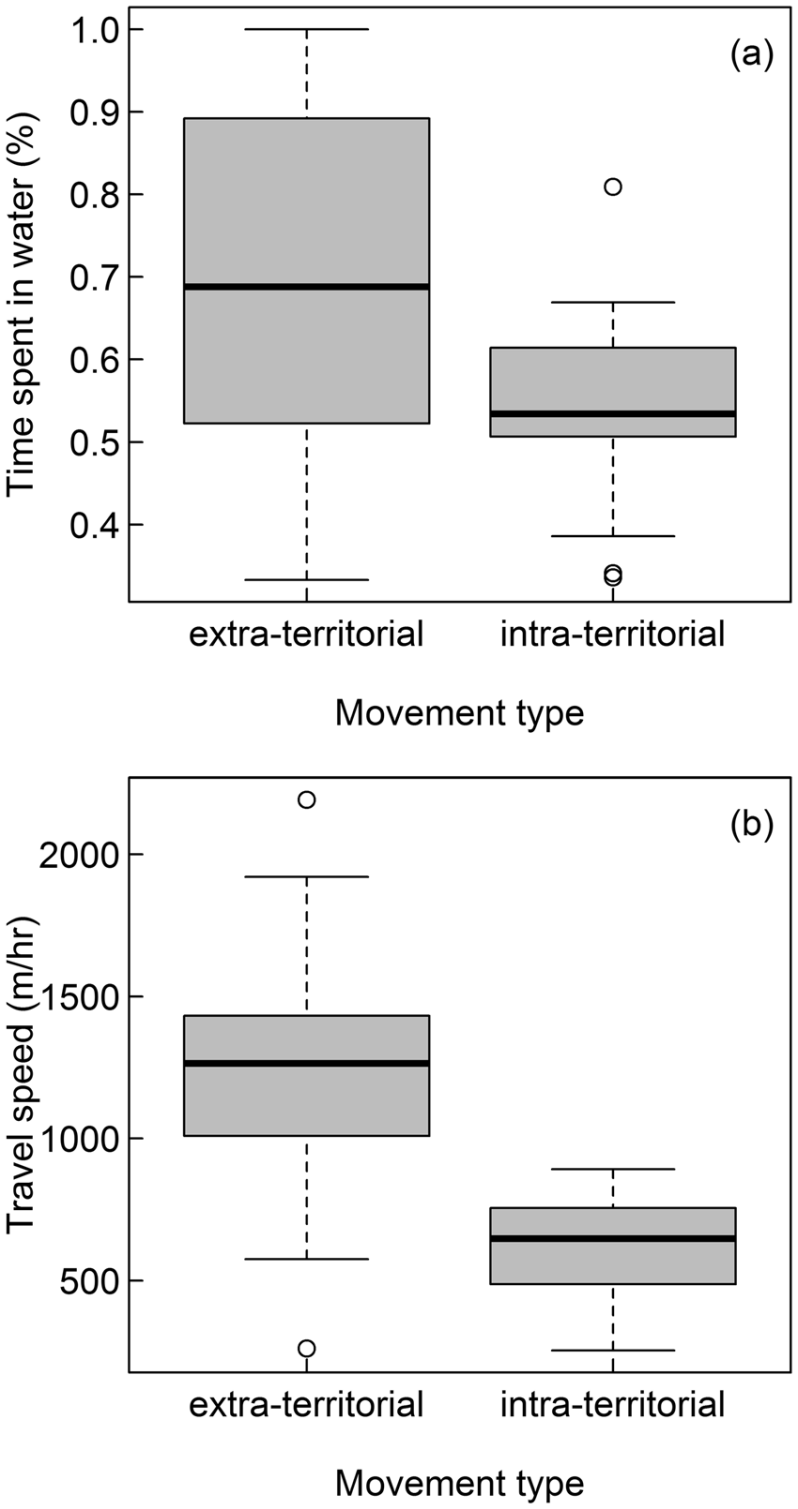
Box plots showing (**a**) the proportion of time spent in water and (**b**) the travel speed (both averaged per individual) for extra-territorial and intra-territorial movements of 25 GPS-tagged Eurasian beavers in southeast Norway (2009–2016). The box plots show the median, 25th and 75th percentile, the range of the data and outliers (dots).

Movement patterns differed between intra-territorial movements and forays (Tables 2 and S1). Beavers spent a larger proportion of time in water when conducting forays compared to intra-territorial movements (Fig. 3a) and during spring compared to autumn (Table 2). Because beavers spend more time in water when on forays, we calculated travel speed for intra-territorial movements only from consecutive water positions, because beavers travel faster in water^39^. Beavers travelled considerably faster during forays compared to intra-territorial movements (Table 2), both when travel speed of intra-territorial movements was calculated from water GPS positions only (Fig. 3b) or from all GPS positions (Table S1). Additionally, beavers travelled faster in larger territories and dominant individuals travelled faster than subordinates (Table 2). When on land, beavers stayed further from the shore when inside their home territory compared to forays (Table 2). In addition, beavers in smaller territories went further from the shore than in larger territories, and subordinates went further from the shore than dominant individuals (Table 2).

**Table 2:** Effect size (β), standard error (SE), and lower (LCI) and upper (UCI) 95% confidence intervals of explanatory variables for (a) the proportion of time spent in water,(b) travel speed, and (c) distance from the shore when on land of 25 GPS-tagged Eurasian beavers in southeast Norway (2009–2016). We performed model averaging of best models (∆AIC_c_ < 4) to estimate the effect size of each variable. Informative parameters are presented in bold.

Variable	β	SE	LCI	UCI
*(a) Proportion of GPS positions in water*				
**Movement type (intra-territorial)**	−**0.666**	**0.133**	−**0.928**	−**0.405**
**Season (spring)**	−**0.403**	**0.146**	−**0.689**	−**0.118**
Status (subordinate)	−0.371	0.192	−0.747	0.005
Territory size (km)	0.086	0.060	−0.032	0.204
Age	−0.010	0.035	−0.079	0.059
*(b) Travel speed (m/hr)*				
**Movement type (intra-territorial)**	−**186.501**	**9.643**	−**205.404**	−**167.597**
**Status (subordinate)**	−**32.371**	**13.646**	−**59.122**	−**5.620**
**Territory size (km)**	**22.907**	**2.481**	**18.043**	**27.771**
Age	−1.824	1.297	−4.368	0.719
Season (spring)	−8.212	8.657	−25.183	8.759
*(c) Distance from the shore when on land (m)*				
**Movement type (intra-territorial)**	**5.170**	**2.603**	**0.066**	**10.273**
**Status (subordinate)**	**10.050**	**4.938**	**0.369**	**19.731**
**Territory size (km)**	−**3.503**	**1.497**	−**6.438**	−**0.568**
Age	0.679	0.798	−0.885	2.243
Season (spring)	1.523	3.640	−5.615	8.660

^*^For extra-territorial movements the distance moved per hour was calculated based on all available GPS positions (there were too few positions to separate for land and water positions); for intra-territorial movements we only used water positions to be conservative (beavers move faster in water and individuals spend more time in water when conducting forays).

### Individual forays

We observed 51 individual forays (43 forays by 20 dominant individuals and 8 forays by 6 subordinates) that ranged between 0.25 and 8.75 hrs (mean ± SD: 1.63 ± 1.62 hrs, median: 1 hr) of an individuals’ active time (between 1900 and 0700 h). Four individuals (2 dominant females and 2 subordinate males) conducted forays that lasted longer than one night, i.e., they spanned over two activity periods (i.e., they spent the daytime away from their own territory). In total (including daytime), these forays lasted between 14.50 and 20.25 hrs (mean ± SD: 16.69 ± 2.61 hrs).

**Figure 4:**
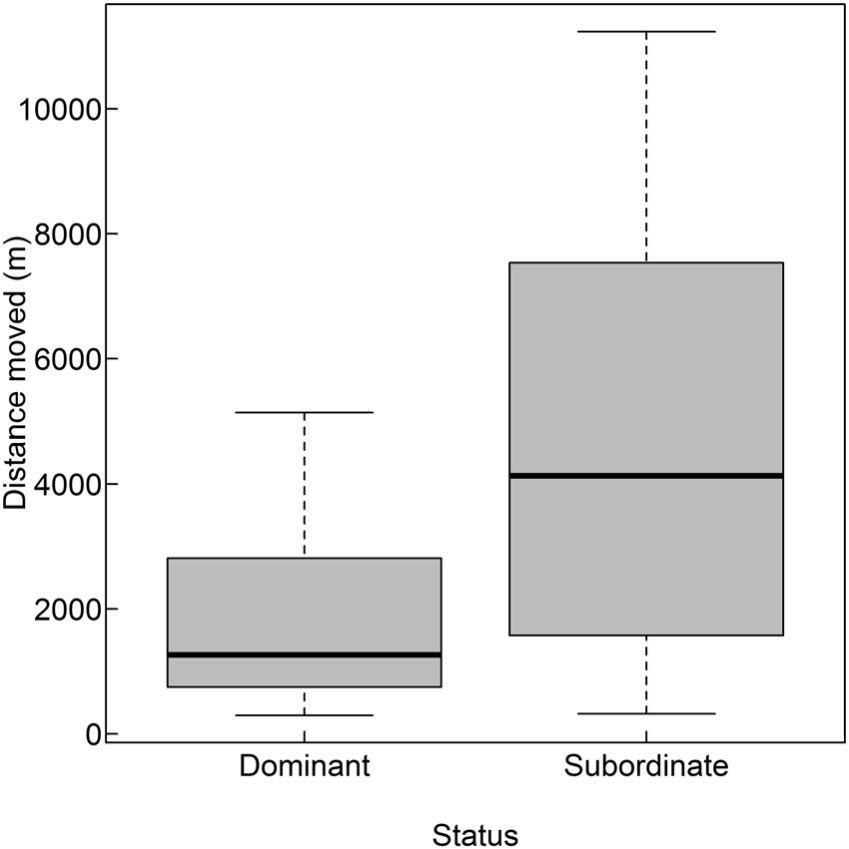
Box plot showing the distance moved (in m) on individual extra-territorial movements separately for dominant (N = 20 individuals, 43 forays) and subordinate (N = 6 individuals, 8 forays) GPS-tagged Eurasian beavers in southeast Norway (2009–2016). Box plots show median values, 25th and 75th percentile, the range of the data and outliers (dot).

The distance moved on individual forays ranged between 298 and 11,237 m (mean ± SD: 2278.80 ± 2212.62 m, median: 1349.07 m), and beavers intruded into 1–5 different territories while conducting forays (mean ± SD: 1.61 ± 1.04, median: 1). The distance moved, duration, and number of intruded territories were highly correlated with each other (r > 0.75, p < 0.001) and were best explained by the status of an individual (Tables 3 and S1). Subordinates moved greater distances during forays compared to dominant individuals (4,756.33 ± 3,862.56 versus 1,817.87 ± 1,398.19 m, Fig. 4) and subordinate forays lasted longer than those by dominant individuals (189.38 ± 162.93 versus 80.93 ± 69.99 min). Further, subordinates intruded into more territories compared to dominants, which mainly intruded only into the adjacent territory (2.75 ± 1.91 versus 1.40 ± 0.62 territories).

**Table 3:** Effect size (β), standard error (SE), and lower (LCI) and upper (UCI) 95% confidence intervals of explanatory variables for (a) the distance moved, (b) the duration, and (c) the number of intruded territories during individual extra-territorial movements (ETM) from 25 GPS-tagged Eurasian beavers in southeast Norway (2009–2016). Informative parameters are presented in bold.

Variable	β	SE	LCI	UCI
*(a) Distance moved (m)*				
**Status (subordinate)**	**2583.300**	**1089.100**	**319.241**	**3932.691**
Age	−100.900	163.600	−396.600	77.967
Territory size (km)	331.900	315.700	−144.764	838.867
Season (spring)	−895.400	701.800	−1562.606	602.485
*(b) Duration (min)*				
**Status (subordinate)**	**123.482**	**52.203**	**18.334**	**228.630**
Age	6.169	8.444	−10.848	23.186
Territory size (km)	12.188	16.032	−20.112	44.487
Season (spring)	−43.817	34.522	−113.369	25.736
*(c) Number of intruded territories*				
**Status (subordinate)**	**0.621**	**0.283**	**0.052**	**1.190**
Territory size (km)	0.137	0.097	−0.057	0.331
Age	−0.033	0.055	−0.142	0.076
Season (spring)	−0.120	0.225	−0.572	0.331

Of the 10 subordinates, 5 were still present in their natal family group when we drafted this manuscript, 3 had dispersed and established a territory, 1 established in its natal territory after the disappearance of its parents, and 1 disappeared for reasons unknown.

## Discussion

We investigated extra-territorial movements (forays) of beavers in southeast Norway. Individuals conducted fewer forays when occupying larger territories and they spend more time in water, travelled faster, and stayed closer to the shore when conducting forays compared to intra-territorial movements. Fewer dominant than subordinate individuals made forays (44% versus 60% of the GPS-tagged individuals). Subordinates generally intruded into multiple territories and moved greater distances than dominants, suggesting that the purpose of these forays is to gain pre-dispersal information as shown in other mammals^3,19,40^.

Independent of their social status, beavers conducted more forays in smaller compared to larger territories and dominant individuals mostly carried out forays only into adjacent territories. Insufficient resource availability in small territories and therefore a need for territory expansion may have motivated these forays. In the same population, Graf, *et al*.^39^ reported that beavers in smaller territories moved further from the shore when on land (i.e., when foraging), possibly due to resource depletion. In addition, Mayer, *et al*.^37^ showed that the duration of territory occupancy was shorter in small as well as large territories, compared to intermediate-sized ones, suggesting that suboptimal sized territories entail costs. In 4 instances, we observed that an individual/pair expanded their territory after the disappearance of one or both dominant individuals of an adjacent territory (unpublished results). Hence, forays might serve as an additional mechanism (apart from scent marking) to rapidly detect changes in neighbouring family groups.

Gosling and McKay^41^ proposed that mammals assess their opponents (or neighbours) by scent matching, i.e., comparing neighbouring scent marks with their own odour. They showed that male house mice (*Mus domesticus*) delayed fighting if scent between opponents matched, indicating that scent marking is used for competitor assessment^41^. Further, Rosell and Bjørkøyli^42^ showed that when beavers were presented with experimental scent mounds of neighbours and strangers, they reacted less aggressive to the scent of the neighbouring individuals. This is known as the dear enemy phenomenon, whereby territory holders respond less aggressively to intrusions by territorial neighbours than by strangers^43^. Consequently, forays could provide an additional mechanism to assess neighbours and to decrease the costs of territory defence, e.g. physical disputes^44^. Forays could also serve to prospect for possibilities to occupy a new territory at a different location. However, this is unlikely in our saturated study population due to a lack of unoccupied territories, and we have only once in 20 years witnessed that a family group moved to a different territory (after the local breeding pair had died due to hunting). Additionally, prospecting could serve to assess future breeding opportunities. In northern wheatears (*Oenanthe oenanthe*), a seasonally territorial bird, individuals that established territories at their previous years prospecting sites, had an increased reproductive success compared to other individuals of the same age^45^, demonstrating that familiarity with an area can be advantageous. Similarly, field sparrows (*Spizella pusilla*) might conduct forays to gain information on future breeding habitat or nest site selection^22^.

In accordance with evidence from Nolet and Rosell^46^, we found that beavers spent less time on land when conducting forays than during intra-territorial movements. Because beavers go on land mostly to forage^28,47^ (apart from scent marking which takes up very little time^47^), it is unlikely that forays serve predominantly as foraging opportunities. When on land, the beavers in our study remained closer to the shore during forays compared to intra-territorial movements, and it has been shown that beavers forage less selectively closer to the shore^29^. Thus, less selective foraging during forays might be a mechanism to increase food intake when risking detection by the territory owner. Independent of the movement type, beavers foraged further from the shore in smaller territories, likely due to resource depletion^37,39^, and subordinates moved further from the shore than dominant individuals, perhaps because they do not invest as much time in territorial activities^48^ and therefore have more time for selective foraging. Other species have also been shown to conduct foraging forays, e.g. wolf packs in Canada conducted more forays to hunt deer when prey densities were low^19^ and female song sparrows (*Melospiza melodia*) likely conducted forays for foraging purposes^49^.

Further, beavers travelled faster during forays than during intra-territorial movements, a finding also reported in red foxes^3^. Faster spatial movements are associated with reduced vigilance to detect predators^50^ or conspecifics^51^ and entail energetic costs^52^. Hence, forays might be costly as suggested by Young and Monfort^6^. The low proportion of forays carried out in our study (on average < 2% of an individuals’ activity time) and the little time spent on land during forays (compared to intra-territorial movements) also indicate that forays could be costly, e.g., when detection by a conspecific ends in a physical dispute. A study in North American beavers showed that conspecific aggression is common with one third of all investigated individuals having injuries^44^. Young and Monfort^6^ reported that male subordinate meerkats (*Suricata suricatta*) had elevated stress levels when conducting forays. This might result in a trade-off between the information and experience gained before dispersal (or the benefits of extra-pair copulations) and the costs of decreased health or even fitness due to stress; and could explain why forays often make up a small proportion of an individuals’ total time budget^16^.

Apart from differences between forays and intra-territorial movements, we found that beavers spent more time on land during spring, possibly to compensate for the loss of body mass during winter^53^. Further, in line with Herr and Rosell^54^ and Graf, *et al*.^39^, we found that beavers travelled faster in larger compared to smaller territories, possibly to be able to patrol the whole territory. Patrolling might also explain why dominants travelled faster compared to subordinates, as they are typically the ones defending the territory^26,48^.

Six of the 10 subordinates conducted forays. They intruded into a larger number of territories and moved greater distances during forays compared to dominant individuals, indicating that these forays were prospecting movements possibly to investigate population density and vacant territories. Mayer, *et al*.^34^ found that subordinate beavers in this study population were more likely to disperse at low population densities. Pre-dispersal forays might provide subordinate individuals with information on population density, vacant territories and/or the condition of territory owners while they profit from remaining safely in the natal family group, i.e. a stay-and-foray tactic rather than becoming a floater^55,56^. Beavers that delayed dispersal had an increased lifetime reproductive success compared to younger dispersers, possibly because they gained information via forays (e.g. on population density^34^) and possibly because they increased their body mass (i.e., competitive ability) while remaining in their natal family group^37^.

Pre-dispersal forays of subordinates have been shown in other mammal^17,19^ and bird^16^ species, and it was suggested that individuals conduct forays in order to gain information on territory occupancy, mate availability, and habitat quality. Debeffe, *et al*.^18^ showed that explorative trips prior to dispersal were more common in future dispersers compared to future philopatric individuals in roe deer (*Capreolus capreolus*), and dispersers were more likely to disperse in the same direction as previously conducted forays. This suggests that dispersal was facilitated by pre-dispersal forays^18^.

In this study, we provide novel evidence that forays by dominant territory owners can be carried out for reasons other than extra-pair copulation or solely foraging, namely to assess their neighbours and possibilities for territory expansion. For dominant territory owners (especially of small territories), neighbour assessment could abet an increase in territory size while reducing conspecific conflict and the costs of territoriality. Subordinates may possibly use forays to gain pre-dispersal information to ultimately increase their dispersal success. As shown by Pärt, *et al*.^45^, forays might have important consequences for the fitness of an individual. However, a direct link between forays and reproductive success remains to be shown in year-round territorial animals.

## Methods

### Study area and data collection

Our study area was located in Telemark County, southeast Norway (Fig. 1) and consisted of 3 rivers (Sauar, Straumen and Gvarv)^54^. The population was at carrying capacity and territories directly bordered each other^39,57^. Beavers have been captured every year since 1998 in spring (March-June) and autumn (August-November), individually marked with a microchip and ear tags^47,58^, and assigned a social status (dominant, subordinate, kit)^59^. Individuals that were captured for the first time as kit or one-year old could be assigned an exact age based on their body mass^60^. Older individuals were assigned a minimum age of 2 years old when captured for the first time with a body mass ≥ 17 kg and ≤ 19.5 kg, and a minimum age of 3 years old with a body mass > 19.5 kg^60^.

From 2009–2016, we equipped 54 individual beavers (15 individuals were tagged multiple times) with a unit consisting of a VHF transmitter (Reptile glue-on, series R1910; Advanced Telemetry Systems, Isanti MN, USA) and a GPS receiver (model G1G 134 A; Sir-track, Havelock North, New Zealand or TGB-317/315GX; Telenax, Playa del Carmen, Mexico). The unit was glued on the lower back (Fig. 1) using a two-component epoxy resin (System Three Resins, Auburn WA, USA)^39^. GPS units recorded GPS positions every 15 min from 1900-0700 h, the beavers’ active time, and were set to sleep during the day when beavers were not active^47^. GPS units recorded between 4 and 22 days of data (mean ± SD: 10.3 ± 3.9 days) and between 102 and 816 GPS positions.

### Ethical note

All trapping and handling procedures were approved by the Norwegian Experimental Animal Board (FOTS id 742, id 2170, 2579, 4384, 6282, 8687) and the Norwegian Directorate for Nature Management (2008/14367 ART-VI-ID, archive code 444.5, 446.15/3, 14415), which also granted permission to conduct fieldwork in our study area. Our study met the ASAB/ABS Guidelines for the treatment of animals in behavioural research and teaching ASAB/ABS,^61^. We captured a total of 54 individuals for this study. None of these individuals were injured during capture and handling, and all were successfully released at the site of capture after handling. No subsequent long-term effects of capture and tagging were observed. All methods were performed in accordance with the relevant guidelines and regulations.

### Data preparation

The capture night was excluded from the analysis to remove possible effects of capture^62^. To correct for imprecise locations, we removed GPS positions with a horizontal dilution of precision value >5 and <4 available satellites (2,537 (10.1%) of 25,000 positions)^63^. We calculated individual territory sizes as river bank length (in km) that was extracted from 95% minimum convex polygons (MCP) of individual GPS positions in ArcMap 10.1 (Esri, Redlands, CA, USA)^39^, but excluding forays as their inclusion would have resulted in a large overestimation of the territory size in some cases. Scent mounds are typically located at territory borders^26^, and we used these marking places to confirm territory borders in the field. Further, in most cases (59 of 73 GPS-tagged beavers) we used GPS data of individuals from adjacent territories, enabling a clear definition of the borders, because adjacent territories did not or only marginally (<10%) overlap^64^. Bank length was chosen as measure of territory size, because beavers typically stay close to the shoreline (both when being on land and in water), and because other methods, like MCP or kernel, could have resulted in an overestimation of territory size due to the inclusion of unused habitat in meandering rivers^39^. A foray was defined as ≥1 GPS position outside of an individual’s territory, if the GPS position(s) was/were 1) >100 m from the individuals’ territory border to account for possible territory overlap^46,64^ and for GPS inaccuracy, and 2) inside a neighbouring or distant family groups’ territory, which was also known from GPS data and from personal observations. Hence, if a GPS position was outside the 95% MCP, but within 100 m from the territory border and not inside a neighbour’s territory, it was not assigned as foray. For each individual, we counted the number of forays. The duration of individual forays was defined from the first to the last consecutive GPS position outside the beavers’ territory. The distance moved (in m) during individual forays was calculated as straight line distance between consecutive foray-GPS positions plus adding the distance between the territory border and the first and last foray-GPS position, respectively. Further, we counted the number of territories a beaver intruded into during individual forays as measure of intruder extent. We defined GPS positions as being in water and on land, respectively, in ArcMap 10.1. Possibly, we wrongly assigned a number of GPS positions to land or water, respectively, due to GPS inaccuracy, erroneous maps and varying water levels. We assume that this was a systematic error, i.e., we erroneously assigned the same proportion of GPS positions to land and water, respectively. To calculate travel speed, we calculated the direct line distance between consecutive GPS positions per hour^39^. There were too few consecutive GPS positions during forays to calculate the travel speed separately for land and water positions (to investigate differences between land and water), however this calculation was possible for intra-territorial movements, because beavers travel faster in water compared to land^39^. For land GPS positions, we calculated the perpendicular distance to the shore in m (termed ‘distance from the shore’).

### Statistical analysis

We investigated the number of forays conducted during the GPS sampling period (dependent variable, N = 54 individuals) using zero-inflated mixed models with a negative-binomial distribution and a logit link using the R package glmmADMB^65^ to account for zero-inflation and overdispersion of the count data. Independent variables were (minimum) age, status (dominant versus subordinate), season (spring versus autumn), the territory size, and the total number of GPS positions (to account for different GPS sampling durations). The beaver ID was included as random effect to control for multiple observations.

To investigate if movement patterns differed between forays and intra-territorial movements, we conducted 3 separate analyses investigating (1) the proportion of time spent in water, i.e., water versus land GPS positions (1 = in water, 0 = on land, 8,733 GPS positions) using a generalized linear mixed model (GLMM) with Bernoulli distribution and a logit link, (2) the travel speed, and (3) the distance from the shore when being on land (3,852 GPS positions) using linear mixed models (LMM) with a Gaussian distribution and an identity link (both latter analyses). In all 3 analyses, the beaver ID was included as random effect, and fixed effects were the movement type (as factor, with intra-territorial movement versus foray), age, status, season, and territory size (N = 25 individuals, 2 were tagged twice).

To examine individual forays, we also conducted 3 separate analyses investigating (1) the distance moved, (2) the duration of individual forays using a LMM with an identity link (both analyses), and (3) the number of intruded territories during individual forays using a GLMM with a Poisson distribution and a log link. Independent variables in all analyses were age, status, season, territory size, and the beaver ID as random effect. During the study period, we additionally conducted scent experiments, simulating a territorial intruder via experimental scent mounds^66^ ^, in revision^, in the territories of 30 GPS tagged beavers included in the present study. Initially, we included the scent experiment (simulated intruder present versus absent) as independent variable, but did not find an effect in any analysis, and thus excluded it from the main analyses to avoid overfitting the models.

For all analyses, we used a set of candidate models including all possible combinations of the fixed effects without interactions. We found no collinearity among independent variables (r < 0.6 in all cases), and variance inflation factors were <3^67^. Model selection was based on Akaike’s Information Criterion corrected for small sample size (AIC_c_)^68^, and was carried out using the R package MuMIn^69^. If ∆AIC_c_ was < 4 in two or more of the most parsimonious models, we performed model averaging^70^. Parameters that included zero within their 95% CI were considered uninformative^71^. All statistical analyses were performed in R 3.2.1^72^.

### Data availability

Appropriate data will be uploaded on Dryad repository upon acceptance.

## Electronic supplementary material

Supplementary tables
