# Supplementary material for: Extra-territorial movements differ between territory holders and subordinates in a large, monogamous rodent
Source: Sci Rep. 2017 Nov 10;7:15261. doi: 10.1038/s41598-017-15540-0 (PMC5681683; doi:10.1038/s41598-017-15540-0)
Supplement: Supplementary file 1 — Supplementary tables [file 41598_2017_15540_MOESM1_ESM.doc]

**Extra-territorial movements differ between territory holders and subordinates in a large, monogamous rodent**

Martin Mayer *^, 1^, Andreas Zedrosser ^1, 2^, Frank Rosell ^1^

^1^ Department of Natural Sciences and Environmental Health, University College of Southeast Norway, Bø i Telemark, Norway

^2^ Department of Integrative Biology, Institute of Wildlife Biology and Game Management, University of Natural Resources and Life Sciences, Vienna, Austria

^*^ Correspondence: M. Mayer, Department of Natural Sciences and Environmental Health, University College of Southeast Norway, 3800 Bø i Telemark, Norway.

Email: martin.mayer@usn.no

**Supplementary tables**

Table S1: The most parsimonious models within ∆AIC_c_ < 4 for the analysis of (a) the number of extra-territorial movements (ETM), (b) the proportion GPS positions on land versus water, the travel speed (calculated from all GPS positions (c) and water positions only (d), respectively), and (e) the distance from the shore when on land of Eurasian beavers, based on data collected between 2009 and 2016 in southeast Norway. Individual ID was included as random effect in all analyses.

| Model | df | logLik | AICc | delta AIC | AIC weight |
| --- | --- | --- | --- | --- | --- |
| *(a) Number of ETMs* |  |  |  |  |  |
| Territory size | 5 | -79.11 | 169.10 | 0.00 | 0.291 |
| Territory size + Season | 6 | -78.38 | 170.00 | 0.92 | 0.184 |
| Territory size + Number of GPS positions | 6 | -78.97 | 171.20 | 2.10 | 0.102 |
| Territory size + Status | 6 | -79.11 | 171.50 | 2.37 | 0.089 |
| Age + Territory size + Season | 7 | -78.13 | 172.00 | 2.86 | 0.070 |
| Age | 5 | -80.70 | 172.30 | 3.17 | 0.060 |
| Territory size + Number of GPS positions + Season | 7 | -78.37 | 172.50 | 3.34 | 0.055 |
| Territory size + Season + Status | 7 | -78.38 | 172.50 | 3.36 | 0.054 |
| Age + Territory size + Status | 7 | -78.46 | 172.60 | 3.52 | 0.050 |
| Age + Territory size + Number of GPS positions | 7 | -78.58 | 172.90 | 3.77 | 0.044 |
|  |  |  |  |  |  |
| *(b) Proportion of GPS positions in water versus on land* |  |  |  |  |  |
| Territory size + Movement type + Season + Status | 6 | -5869.99 | 11752.00 | 0.00 | 0.271 |
| Movement type + Season + Status | 5 | -5871.13 | 11752.30 | 0.28 | 0.235 |
| Age + Territory size + Movement type + Season + Status | 7 | -5869.63 | 11753.30 | 1.28 | 0.143 |
| Movement type + Season | 4 | -5872.68 | 11753.40 | 1.37 | 0.137 |
| Age + Movement type + Season + Status | 6 | -5871.13 | 11754.30 | 2.27 | 0.087 |
| Territory size + Movement type + Season | 5 | -5872.42 | 11754.80 | 2.85 | 0.065 |
| Age + Movement type + Season | 5 | -5872.45 | 11754.90 | 2.91 | 0.063 |
|  |  |  |  |  |  |
| *(c) Travel speed (m/hr) calculated from all GPS positions* |  |  |  |  |  |
| Age + Movement type + Status + Season + Territory size | 8 | -107344.50 | 214705.10 | 0.00 | 1.000 |
|  |  |  |  |  |  |
| *(d) Travel speed (m/hr) calculated from water GPS positions only* |  |  |  |  |  |
| Age + Movement type + Status + Season + Territory size | 8 | -35198.71 | 70413.40 | 0.00 | 0.762 |
| Movement type + Status + Season + Territory size | 7 | -35200.88 | 70415.80 | 2.33 | 0.238 |
|  |  |  |  |  |  |
| *(e) Distance from the shore when on land (m)* |  |  |  |  |  |
| Age + Movement type + Status + Season + Territory size | 8 | -17678.58 | 35373.20 | 0.00 | 0.399 |
| Movement type + Status + Season + Territory size | 7 | -17679.62 | 35373.30 | 0.08 | 0.385 |
| Age + Movement type + Status + Territory size | 7 | -17680.84 | 35375.70 | 2.52 | 0.114 |
| Movement type + Status + Territory size | 6 | -17681.95 | 35375.90 | 2.73 | 0.102 |

Table S2: The most parsimonious models within ∆AIC_c_ < 4 for the analysis of (a) the distance moved during individual extra-territorial movements, (b) the duration of individual forays, and (c) the number of territories intruded during individual forays of Eurasian beavers, based on data collected between 2009 and 2016 in southeast Norway. Individual ID was included as random effect in all analyses.

| Model | df | logLik | AICc | delta AIC | AIC weight |
| --- | --- | --- | --- | --- | --- |
| *(a) Distance moved during individual ETMs* |  |  |  |  |  |
| Age + Season + Status + Territory size | 7 | -421.98 | 860.60 | 0.00 | 1.000 |
|  |  |  |  |  |  |
| *(b) Duration of individual ETMs* |  |  |  |  |  |
| Age + Season + Status + Territory size | 7 | -279.61 | 575.80 | 0.00 | 0.877 |
| Season + Status + Territory size | 6 | -282.91 | 579.70 | 3.92 | 0.123 |
|  |  |  |  |  |  |
| *(c) Number of territories intruded into during individual ETMs* |  |  |  |  |  |
| Status | 3 | -68.51 | 143.50 | 0.00 | 0.310 |
| Status + Territory size | 4 | -67.91 | 144.70 | 1.17 | 0.173 |
| Status + Season | 4 | -68.38 | 145.60 | 2.10 | 0.108 |
| Territory size | 3 | -69.62 | 145.70 | 2.23 | 0.102 |
| Age + Status | 4 | -68.50 | 145.90 | 2.34 | 0.096 |
| Age + Territory size | 4 | -68.52 | 145.90 | 2.40 | 0.094 |
| Status + Season + Territory size | 5 | -67.74 | 146.80 | 3.28 | 0.060 |
| Age + Status + Territory size | 5 | -67.77 | 146.90 | 3.35 | 0.058 |
